# Global analysis of cis-natural antisense transcripts and their heat-responsive nat-siRNAs in *Brassica rapa*

**DOI:** 10.1186/1471-2229-13-208

**Published:** 2013-12-10

**Authors:** Xiang Yu, Jun Yang, Xiaorong Li, Xuxin Liu, Chuanbao Sun, Feijie Wu, Yuke He

**Affiliations:** 1 National Key Laboratory of Plant Molecular Genetics, Shanghai Institute of Plant Physiology and Ecology, Shanghai Institutes for Biological Sciences, Chinese Academy of Sciences, 300 Fenglin Road, Shanghai 200032, China

**Keywords:** cis-NATs, nat-siRNAs, Heat response, Genomic comparison, *Brassica rapa*

## Abstract

**Background:**

*Brassica rapa* includes several important leaf vegetable crops whose production is often damaged by high temperature. Cis-natural antisense transcripts (cis-NATs) and *cis*-NATs-derived small interfering RNAs (nat-siRNAs) play important roles in plant development and stress responses. However, genome-wide cis-NATs in *B. rapa* are not known. The NATs and nat-siRNAs that respond to heat stress have never been well studied in *B. rapa*. Here, we took advantage of RNA-seq and small RNA (sRNA) deep sequencing technology to identify cis-NATs and heat responsive nat-siRNAs in *B. rapa*.

**Results:**

Analyses of four RNA sequencing datasets revealed 1031 *cis*-NATs *B. rapa* ssp. *chinensis* cv Wut and *B. rapa* ssp. *pekinensis* cv. Bre. Based on sequence homology between *Arabidopsis thaliana* and *B. rapa*, 303 conserved *cis*-NATs in *B. rapa* were found to correspond to 280 cis-NATs in *Arabidopsi*s; the remaining 728 novel *cis*-NATs were identified as *Brassica*-specific ones. Using six sRNA libraries, 4846 nat-siRNAs derived from 150 *cis*-NATs were detected. Differential expression analysis revealed that nat-siRNAs derived from 12 cis-NATs were responsive to heat stress, and most of them showed strand bias. Real-time PCR indicated that most of the transcripts generating heat-responsive nat-siRNAs were upregulated under heat stress, while the transcripts from the opposite strands of the same loci were downregulated.

**Conclusions:**

Our results provide the first subsets of genome-wide cis-NATs and heat-responsive nat-siRNAs in *B. rapa*; these sRNAs are potentially useful for the genetic improvement of heat tolerance in *B. rapa* and other crops.

## Background

Natural antisense transcripts (NATs) are endogenous RNA molecules that exhibit partial or complete complementarity to other transcripts. Cis-NATs are transcribed from the opposite DNA strand as their sense transcripts from the same genomic loci. Genome-wide analyses have revealed that cis-NATs are widespread in eukaryotes [1,2]. In animal and plant genomes, l7-30% of the genes encode complementary cis-NATs [3-7]. In animals, NATs are involved in alternative splicing, DNA methylation, RNA editing and genomic imprinting [8-11]. In plants, several cis-NATs take part in gene regulatory events through cis-NAT-derived small interferering RNAs (nat-siRNAs) [12,13]. Cis-NATs have been identified on genome-wide scale in Arabidopsis and rice [6,14,15]. These cis-NATs produce nat-siRNAs in the regions overlapping with sense transcripts and the nat-siRNAs exhibit strand-specificity (strand bias) [16].

Small non-coding RNAs are well known as an important regulatory factor in gene networks, and are widely involved in different development stages and stress responses. Based on the genomic origins of their precursors, sRNAs can be divided into four categories: microRNAs (miRNAs), trans-acting small interfering RNAs (ta-siRNAs), NAT siRNAs (nat-siRNAs), and repeat-associated siRNAs [17]. Among these four categories, the biogenesis and function of miRNA are best understood [18,19], and they participate in a broad range of developmental and stress response events [20,21]. Most miRNAs are derived from intergenic regions, although some of them are processed from introns of protein-coding genes [22]. Nat-siRNAs that participate in stress responses at specific development stage have been discovered in recent years. The first identified salt-induced nat-siRNAs were derived from the antisense overlapping gene pair of Delta (1)-pyrroline-5-carboxylate dehydrogenase (P5CDH), a stress-related gene (SRO5); 24-nt (nucleotides) siRNAs are formed by a biogenesis pathway dependent on Dicer-like 2 (DCL2), while a phase for the subsequent generation of 21-nt siRNAs are established by DCL1 [12]. In 2006, a pathogen-induced nat-siRNA was found and its biogenesis required DCL1 [13]. A plant sperm-specific nat-siRNA plays an important role in controlling sperm function during double fertilization [23]. However, the general biogenesis process and regulatory mechanisms of NATs and nat-siRNAs are still largely unclear.

*Brassica rapa* comprises several important leaf vegetable crops, and its genome is considered to be mesohexaploid and derived from a triplicated diploid ancestral genome that was closely related to the *A. thaliana* genome [24,25]. Recently, many conserved and novel miRNAs have been identified in *B. rapa* through sRNA deep sequencing [26,27]. However, the genome-wide cis-NATs and heat-responsive nat-siRNAs in *B. rapa* are still unknown. Leaf vegetable crops are much more sensitive to high temperature than fruit vegetable crops. Some of *B. rapa* varieties are highly sensitive to high temperature, and thus have very circumscribed growing seasons.

The common symptoms of heat sensitivity (also known as heat intolerance) are leaf etiolation and wilting [28]. Under too-warm conditions, many *B. rapa* varieties exhibit these traits and stop growing. In recent years, a great deal of attention has been paid to elucidating the mechanisms of heat-sensitivity in *B. rapa*. High temperature suppresses the production of some chloroplast-specific small RNAs that may function in transcriptional or post-transcriptional regulation. Using RNA and small RNA sequencing datasets, we detected the genome-wide cis-NATs and heat-responsive nat-siRNAs in *B. rapa*.

## Results

### Gene models and cis-NATs in *B. rapa*

In the *B. rapa* genome v1.1, Wang et al. annotated 39786 gene models, including only coding sequences (CDSs) without un-translated regions (UTRs) [29]. To identify the genome-wide cis-NATs in *B. rapa*, we performed the RNA sequencing using RNA isolated from *B. rapa* ssp. *chinensis* cv. Wut, a heat-sensitive variety, and *B. rapa* ssp. *pekinensis* cv. Bre, a less heat-sensitive variety. In the seedlings, 2.77 million and 2.56 million reads were present in Bre and Wut, respectively; and the inflorescence apices yielded 1.80 million and 2.69 million reads in Bre and Wut, respectively (Additional file 1: Table S1).

**Figure 1:**
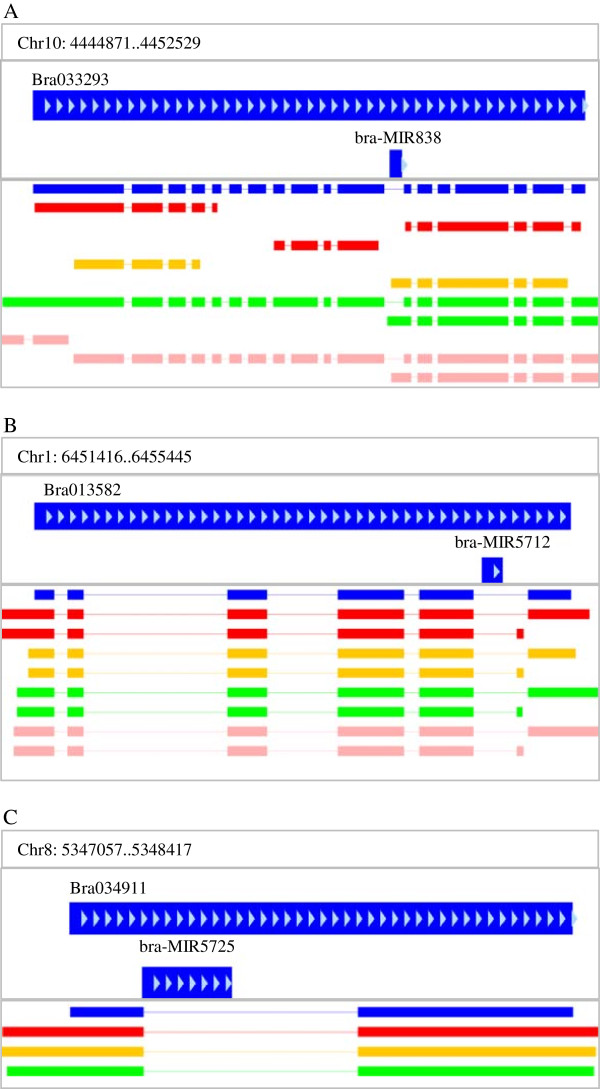
**Transcript models of host genes from which intronic miRNAs were derived*. *(A)** Bra-MIR838 from the 11^th^ intron of Bra033293. **(B)** Bra-MIR5712 from the 5^th^ intron of Bra013582. **(C)** Bra-MIR5725 from the sole intron of Bra034911. Blue rectangles indicate the original coding sequence models without untranslated regions; red rectangles indicate *Brassica rapa* ssp. *pekinensis* cv. Bre seedling; orange rectangles indicate *B. rapa* ssp. *chinensis* cv. Wut seedling; green rectangles indicate cv. Bre inflorescence apex; pink rectangles indicate cv. Wut inflorescence apex.

Using the Tophat-Cufflink pipeline [30], the transcripts detected from seedlings and inflorescence apices of Bre and Wut were assembled (Table 1). After alignment with the annotated genes of *B. rapa*, 24099 and 24948 gene models with the longest transcripts were extracted for Bre and Wut, respectively. In addition to the miRNAs identified from Wut in a previous study [26], 155 conserved miRNA precursors (pre-miRNAs) and 19 *Brassica*-specific pre-miRNAs were newly annotated (Additional file 2: Table S2). Interestingly, four intronic miRNAs were derived from their host genes and Bra-MIR156B-2 originated from the 10^th^ intron of its host gene Bra024030, while Bra-MIR838 from the 11^th^ intron of its host gene Bra033293, the homolog of *AtDCL1* in *Arabidopsis* (Figure 1A). Furthermore, two *Brassica*-speicific intronic miRNAs were discovered: Bra-MIR5712 was processed from the 5^th^ intron of Bra013582 and Bra-MIR5725 from the sole intron of Bra034911 (Figure 1B, C).

**Table 1:** **Overview of *B. rapa *** **transcripts assembled through the Tophat-Cufflink pipeline**

	**Transcripts**	**Gene model**	**Gene model union**	**Novel genes**	**AS transcripts**	**AS events**
Bre_Seedling	30485	22521	24099	1542	3527	34012
Bre_IA	27916	23135		1924	6968	34884
Wut_Seedling	31242	22407	24948	1700	3390	34632
Wut_IA	28331	23053		2210	8122	36453

IA: inflorescence apices.

AS: Alternative splicing.

**Figure 2:**
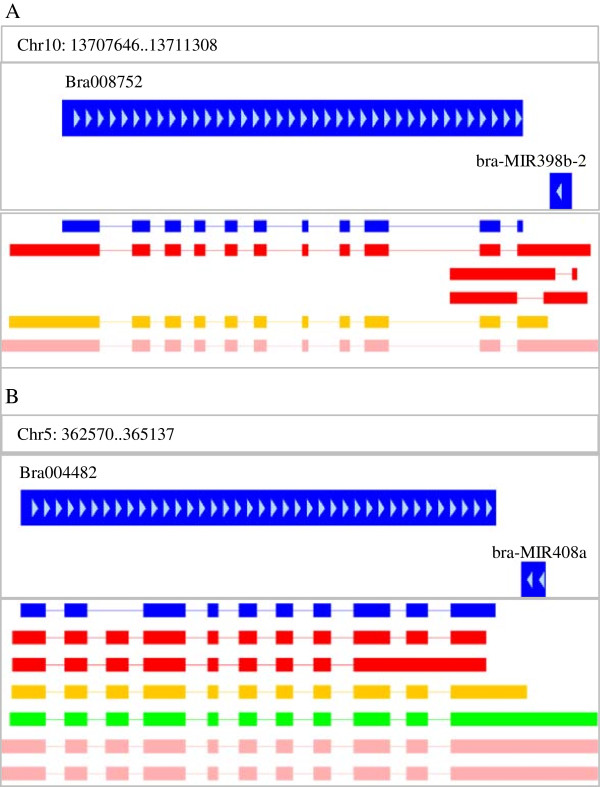
**Cis-NAT pairs that are miRNA precursor and coding sequences. (A)** Cis-NAT pair of Bra008752 and bra-MIR398b-2 and assembled transcript models of Bra008752. **(B)** Cis-NAT pairs of Bra004482 and bra-MIR408a and assembled transcript models of Bra004482. Blue rectangles indicate the original coding sequence model without untranslated regions; red rectangles indicate *Brassica rapa* ssp. *pekinensis* cv. Bre seedling; orange rectangles indicate in *B. rapa* ssp. *chinensis* cv. Wut seedling; green rectangles indicate cv. Bre inflorescence apex; pink rectangles indicate cv. Wut inflorescence apex.

To select cis-NATs, we searched for gene pairs that overlapped by more than 25 nt and were transcribed from the opposite DNA strands. We detected 721 and 648 pairs of cis-NATs in Bre and Wut, respectively (Table 2). The cis-NATs were categorized into three types: convergent (3′ end overlap), divergent (5′ end overlap) and enclosed (one transcript encompassed the other transcript). Most of cis-NATs in *B. rapa* were convergent-orientated (597 and 544 cis-NATs in Bre and Wut, respectively), consistent with the reports in *Arabidopsis*. Of these cis-NATs, 450 were identified in both Bre and Wut and there were a total of 1031 cis-NATs in the two varieties. Eight cis-NATs were coincidently the precursors of miR162, miR167, miR171, miR172, miR398 and miR408 (Table 3). For example, Bra-MIR398b-2 overlapped with Bra008752 and Bra-MIR408a with Bra004482 (Figure 2). In this case, some sRNAs might be derived from miRNA precursor rather than cis-NATs. Therefore, the cis-NATs that overlapped with miRNA precursors were excluded from the cis-NAT database.

**Table 2:** **Different types of cis-NATs in *B. rapa***

	**Convergent**	**Divergent**	**Enclosed**	**Total**	**Nat-siRNA-producing**	**Strand bias**
Bre	597	50	74	721	57 (7.91%)	42
Wut	544	42	62	648	111 (17.13%)	66
Shared^a^	271	23	32	338	18 (5.33%)	10
Specific in Bre	326	27	42	383	39 (10.18%)	32
Specific in Wut	273	19	30	310	93 (30.00%)	56
General in *B. rapa*^b^	870	69	104	1031	150 (14.55%)	98

^a^Intersection between Bre and Wut cis-NATs.

^b^Union between Bre and Wut cis-NATs.

**Table 3:** List of pri-miRNA in cis-NATs

**Pri-miRNA**	**Paired genes**
bra-MIR162a	Bra028668
bra-MIR167d	Bra023163
bra-MIR171a-2	Bra012855
bra-MIR171a-3	Bra036812
bra-MIR172a-1	Bra000498
bra-MIR408a	Bra004482
bra-MIR398b-1	Bra006261
bra-MIR398b-2	Bra008752

### Conserved and novel cis-NATs in *B. rapa*

Using the Arabidopsis genome annotation (TAIR 10 version), we detected 1587 pairs of cis-NAT with overlapping region that was more than 25-nt in Arabidopsis. Based on the homologous gene annotation between *Arabidopsis* and *Brassica*[29], we found that 303 conserved cis-NATs in *B. rapa* corresponded to 280 cis-NATs in *Arabidopsis* (Additional file 3: Table S5). 21 cis-NATs in *Arabidopsis* had two copies cis-NATs in *B. rapa*, while cis-NAT pair of AT3G12250/AT3G12260 had 3 copies of cis-NATs in *B. rapa*. Thus, 728 novel cis-NATs were *Brassica*-specific. Among 150 cis-NATs that generated nat-siRNAs in *B. rapa*, 47 were conserved in *Arabidopsis*, revealing that approximately 100 cis-NATs produced nat-siRNAs specifically in *B. rapa*. In *Arabidopsis*, Zhang et al. identified 84 cis-NATs that give rise to nat-siRNAs from 21 sRNA libraries [16]. 9 of them existed in *B. rapa*.

### Nat-siRNAs in overlapping region of cis-NATs

To survey genome-wide nat-siRNAs derived from cis-NATs, we constructed and data mined six sRNA libraries. Two were prepared from the seedlings of Bre exposed to biotic stress and four from the seedling of Wut exposed to abiotic stress. For biotic stress, Bre plants were either uninfected (control) or infected by the bacterial strain *Erwinia carotovora* ssp. *carotovora* (Ecc), which causes soft rot disease [31]. Wut plants were exposed to either 22°C (Normal temperature, NT) or 46°C (high temperature, HT) for 1 h, with two replicates (NT1, HT1, NT2, and HT2) [26,28]. In total, we obtained 4.49 million and 4.09 million sRNA reads in the control and bacteria-infected Bre plants, respectively, and 14.67 million, 12.77 million, 11.25 million and 14.61 million reads in HT1, HT1, NT2, and NT2 Wut seedlings, respectively. The abundance of each dataset was normalized to RP10M (reads per 10 million).

**Figure 3:**
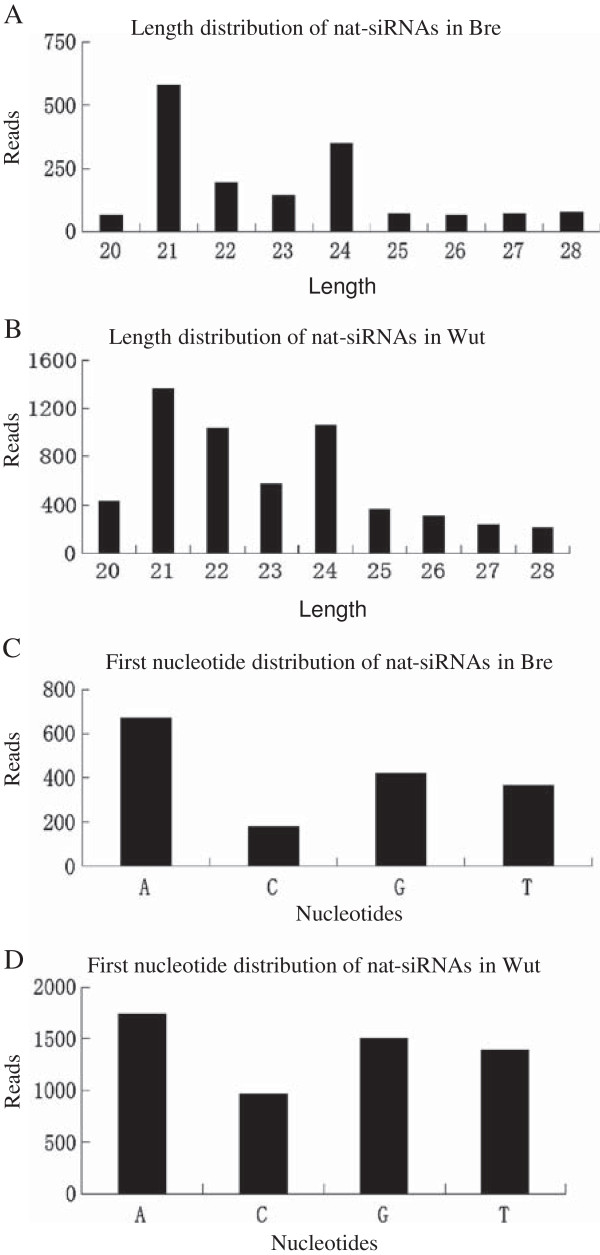
**Length distribution and first-nucleotide distribution of nat-siRNA in Bre and Wut. (A)** Length distribution of nat-siRNAs in *Brassica rapa* ssp. *pekinensis* cv. Bre; **(B)** Length distribution of nat-siRNAs in *B. rapa* ssp. *chinensis* cv. Wut; **(C)** First-nucleotide distribution of nat-siRNAs in cv. Bre; **(D)** First-nucleotide distribution of nat-siRNAs in cv. Wut.

We searched for sRNAs with more than 5 reads perfectly matched with 20–28 nt of the overlapping regions in the cis-NATs, and found 57 pairs of nat-siRNAs in Bre and 111 pairs in Wut (Table 2). In total, 1641 reads corresponding to 533 unique nat-siRNAs and 5623 reads corresponding to 4313 unique nat-siRNAs were detected in overlapping regions of Bre and Wut cis-NATs, respectively. The nat-siRNAs had a length bias of 21-nt, consistent with those in *Oryza sativa*[14] (Figure 3A-B). The nat-siRNAs exhibited a bias for adenine in both the two varieties (Figure 3C-D). We analyzed the cis-NATs that generate nat-siRNAs with strand bias. By calculating the ratios of nat-siRNA reads on forward strand versus reverse strands, we found that the nat-siRNAs from 42 Bre cis-NATs and 66 Wut cis-NATs exhibited strand bias, with ratios greater than two-fold and *p*-value <0.01 (Additional file 4: Table S3, Additional file 5: Table S4). Among 150 pairs of cis-NAT generating nat-siRNAs, 98 exhibited strand bias (Table 2).

### Nat-siRNAs responsive to heat stress

**Figure 4:**
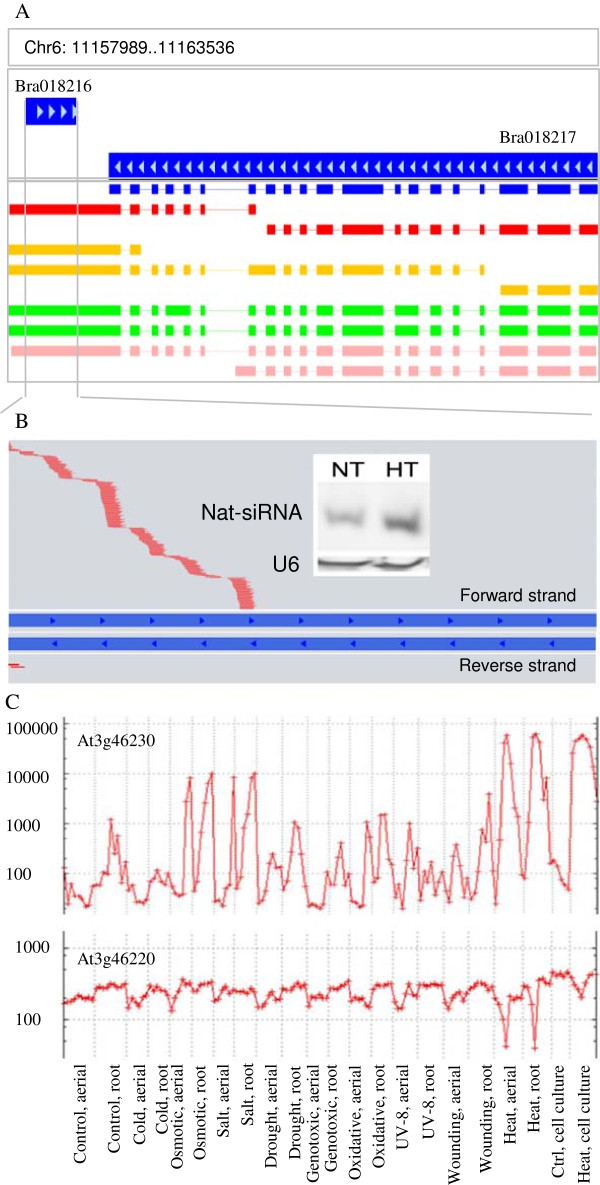
**Heat-responsive nat-siRNAs derived from Bra018217/Bra018216 cis-NAT pair and expression of their homologs in Arabidopsis. (A)** Assembled transcript models of Bra018217 in four RNA-seq datasets. Blue rectangles indicate the original coding sequence model without untranslated regions; red rectangles indicate *Brassica rapa* ssp. *pekinensis* cv. Bre seedling; orange rectangles indicate in *B. rapa* ssp. *chinensis* cv. Wut seedling; green rectangles indicate cv. Bre inflorescence apex; pink rectangles indicate cv. Wut inflorescence apex. **(B)** Nat-siRNAs were derived from the strands of Bra018216 (top of gene pairs) or of Bra018217 (bottom). The red lines indicate heat-induced nat-siRNAs in high-temperature libraries, and the black one indicates nat-siRNAs in normal-tmperature libraries. Top right corner is the northern bolt result of nat-siRNAs that derived from the strand of Bra018216 under normal treatment (NT) and heat treatment (HT). **(C)** Expression of At3G46230, a homolog of Bra018216, and At3G46220, a homolog of Bra018217, under abiotic stress (AtGenExpress Visualization Tool).

To identify heat-responsive nat-siRNAs in *B. rapa*, we compared the abundance of nat-siRNAs produced at NT and HT in Wut (Additional file 6: Table S6). We found that the nat-siRNAs from six cis-NATs increased under heat stress, while those from six decreased (the threshold of 2-fold change and *p*-value <0.01; Table 4). Among them, four cis-NATs that gave rise to nat-siRNAs were conserved in *Arabidopsis*. Heat-responsive nat-siRNAs from 10 cis-NATs exhibited strand bias, implying that they targeted the genes in the opposite strands of cis-NATs. The cis-NAT pair Bra018216/Bra018217 belonged to the enclosed type; the entire transcript of Bra018216 was in the opposite strand of Bra018217 3′-UTR (Figure 4A). Nat-siRNAs from Bra018216/Bra018217 pair were induced specifically by heat, and nat-siRNAs derived from the strand of Bra018216 were most abundant (Figure 4B). Northern blotting confirmed that the abundance of nat-siRNAs derived from Bra018216 was much higher at HT than at NT (Figure 4B).

**Table 4:** The ratios of high-temperature cis-nat-siRNAs to normal-temperature ones derived from cis-NATs

**cis-NAT Pairs**		**HT1/NT1**	***p*-value**	**HT2/NT2**	***p*-value**	**FR/RR**	**Putative target genes**
Bra018111	Bra018112	0.04	0	0.26	0.000001	0.09	Bra018111
Bra024428	Bra024429	0.04	0	0.17	0.000001	0.00	Bra024428
Bra023553	Bra023554	0.06	0.000018	0.2	0.002686	INF	Bra023554
Bra027167	Bra027168	0.08	0.000427	0.2	0.002686	0.18	Bra027167
Bra013578	Bra013579 (C)	0.31	0.000167	0.14	0.00751	0.05	Bra013578
Bra012677	Bra012676	0.38	0.002245	0.5	0.009327	0.17	Bra012677
Bra030183	Bra030182	2.18	0	2	0	1.93	
Bra002999	Bra002998 (C)	4.05	0	6.17	0	5.68	Bra002998
Bra000460	Bra000461	4.33	0.004272	2.67	0.004272	0.80	Bra000460
Bra027180	Bra027181 (C)	5.86	0	17	0	0.87	
Bra040444	Bra040445	11	0.001465	7	0.001465	0.05	Bra040444
Bra018216	Bra018217 (C)	INF^a^	0	INF	0	247.50	Bra018217

The cis-NAT pair with (C) is conserved in Arabidopsis.

FR: nat-siRNA reads derived from the forward strands.

RR: nat-siRNA reads derived from the reverse strands.

^a^INF means that the total reads of NT1 or NT2 is 0.

**Figure 5:**
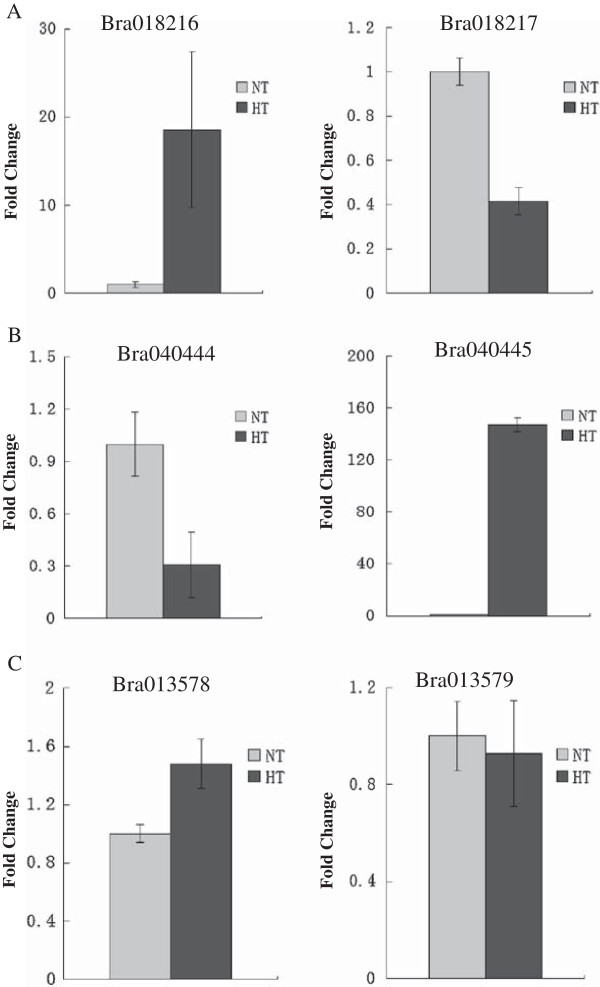
**Relative expression of cis-NATs at high temperature detected by real-time PCR. (A)** Expression of Bra028216 and Bra028217 in normal temperature (NT) and high-temeperature (HT) seedlings. **(B)** Expression of Bra040444 and Bra040445 in NT and HT seedlings. **(C)** Expression of Bra013578 and Bra013579 in NT and HT seedlings.

**Figure 6:**
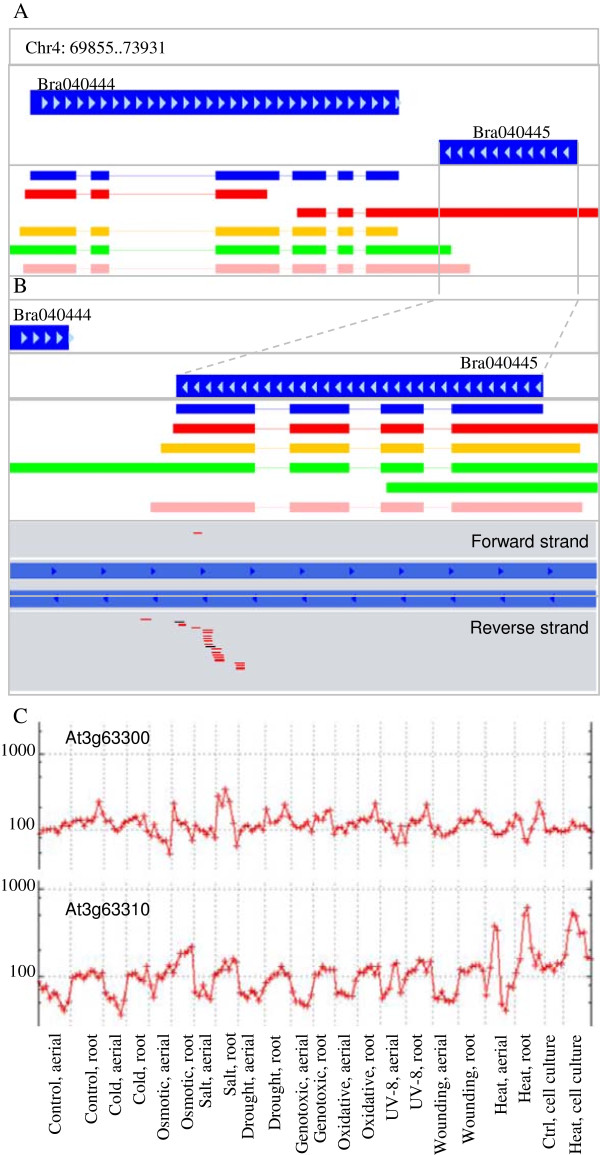
**Heat-responsive nat-siRNAs derived from Bra040444/ Bra040445 cis-NAT pair and expression of their homologs in Arabidopsis. (A)** Assembled transcript models of Bra040444 in four RNA-seq datasets. **(B)** Assembled transcript models of Bra040445 in four RNA-seq datasets. Nat-siRNAs were derived from the strands of Bra040444 (top of gene pairs), or of Bra040445 (bottom). The red lines indicate heat-induced nat-siRNAs in high-temperature libraries, and black ones indicate nat-siRNA in normal-temperature libraries. **(C)**  Expression of AT3G63300, a homolog of Bra040444, and AT3G63310,a homolog of Bra040445, under abiotic stress (AtGenExpress Visualization Tool). Blue rectangles indicate the original coding sequence model without untranslated regions; red rectangles indicate *Brassica rapa* ssp. *pekinensis* cv. Bre seedling; orange rectangles indicate in *B. rapa* ssp. *chinensis* cv. Wut seedling; green rectangles indicate cv. Bre inflorescence apex; pink rectangles indicate cv. Wut inflorescence apex.

To define the relationship between cis-NATs and nat-siRNAs at high temperature, we performed real-time PCR of both Bra018216 and Bra018217 transcripts. At high temperature, Bra018216 levels sharply increased, whereas those of Bra018217 decreased (Figure 5A). These changes were concurrent with an increase in abundance of the heat-induced nat-siRNAs derived from Bra018216. The homologous cis-NAT pair of Bra018216/Bra018217 in Arabidopsis is AT3G46230/AT3G46220. AT3G46230 encodes a class I small heat-shock protein (sHSP) HSP17.4 that shows a role in heat resistance. We compared the relative expressions of these two genes using the abiotic stress data of AtGenExpress [32]. Under the condition of 38°C for 3 h, AT3G46220 was specifically repressed, while AT3G46230 was sharply induced in both seedlings and roots (Figure 4C). Thus, the homologous cis-NAT pair AT3G46230/ AT3G46220 also generates nat-siRNAs responsive to heat stress. The nat-siRNAs from the Bra040444/ Bra040445 cis-NAT pair were induced by heat stress. Similarly, the expression of Bra040445 was also induced by heat stress, while Bra040444 was repressed (Figure 5B). The 3′-UTR of Bra040445 generated many more nat-siRNAs than the coding region (Figure 6A-B). Like Bra040445, the homologous AT3G63310 in Arabidopsis was induced under heat stress. Nevertheless, AT3G63300 and AT3G63310 did not overlap to form cis-NATs (Figure 6C). AT3G63300 was insensitive to heat, while its homolog Bra040444 was repressed by the nat-siRNAs. Real-time PCR showed that Bra013578 was up-regulated in Wut seedlings, while most nat-siRNAs derived from the strands of Bra013579 decreased under heat stress (Figure 6C, Table 4).

## Discussion

### More cis-NATs and nat-siRNAs in *B. rapa* after genome triplication

In this study, we globally identified 1031 cis-NATs and 4846 nat-siRNAs derived from 150 cis-NATs in two varieties of *B. rapa.* The nat-siRNAs are thought to be processed from overlapping regions of transcribed cis-NATs. The diploid ancestral genome of mesohexaploid *B. rapa* was closely related to the *A. thaliana* genome [25]. Comparison of cis-NATs between these two species showed that 303 conserved cis-NATs in *B. rapa* corresponded to 280 cis-NATs in Arabidopsis. 22 Arabidopsis cis-NATs have two or three copies of homologous cis-NATs in *B. rapa*. In *B. rapa*, cis-NATs with more than 2 copies would crosstalk with each other and form trans-NATs. Based on pairwise alignments of transcripts, 47 cis-NATs that produce nat-siRNAs in *B. rapa* are conserved in *Arabidopsis*, implying that the two thirds of the cis-NATs in *B. rapa* generate specific nat-siRNAs. When nat-siRNAs are examined in other subspecies and varieties of *B. rapa*, more nat-siRNAs from conserved cis-NATs will be detected. The interaction of multiple copies of cis-NATs must be more complex, but should not bring chaos, because the individual variants with chaos are died out during evolution.

The nat-siRNAs in coding sequences are more conserved than those in other regions as described in *Arabidopsis lyrata*[33]. The roles of conserved cis-NATs and nat-siRNAs may be general in development events or response to environment stimuli of plants, whereas those of the novel cis-NATs in *B. rapa* should be specific. Interestingly, some *Brassica*-specific cis-NATs and nat-siRNAs are more than two. These overlapping-sequences are differentiated over time, since many SNPs has been identified in different *Brassica* crops. Possibly, two copies of an Arabidopsis cis-NAT in *B. rapa* occurs when they are trans-NATs (at different loci). Our study is focused on cis-NATs rather than trans-NATs, which are more complex. It is not known whether cis-NATs and trans-NATs are different in regulation of target genes.

Previous work in *Arabidopsis* indicated that nat-siRNAs could target and cleave one of the cis-NAT at the posttranscriptional level [12]. Heat-induced nat-siRNAs generated from an up-regulated transcript may function in repressing its targets, which should be cleared under heat stress. The nat-siRNAs could be generated from both cis-NATs and trans-NATs, and targets to the transcripts with high sequence complimentarity. Because *B. rapa* has multiple homolog genes, T-DNA insertion could knock-out only one copy. However, over-expression of a single cis-NAT could knock-down several homologous targets, thus, suggesting potential application of nat-siRNAs from cis-NATs for improving heat-resistance.

### Strand bias in heat-responsive cis-NATs and nat-siRNAs

Nat-siRNAs has been shown to response to environment stimuli such as salt stress and biotic stress [34]. In plants, nat-siRNASRO5 is induced by salinity [12], while nat-siRNAATGB2 only accumulates in response to bacterial pathogen infection [13]. However, which nat-siRNAs are involved in heat stress is largely unknown, even in *Arabidopsis*. We identified 12 cis-NATs that respond to heat stress, with strand bias.

The nat-siRNAs derived from four conserved cis-NATs are conserved in Arabidopsis. Like the salt-induced gene *SRO5* which forms a double helix with its cis-NAT pair *P5CDH*[12], Bra018216 is induced by heat stress, and is complementary to Bra018217 to produce dispersed siRNAs. However, the length of nat-siRNAs from Bra018216/Bra018217 ranged from 20–28 nt rather than 21 or 24 nt. Nat-siRNAs of 20–22 nt are generated by DCL1, whereas the 23–28-nt nat-siRNAs are DCL3-dependent [16]. The nat-siRNAs derived from Bra018216 may repress Bra018217 expression. Whether over-expression of Bra018216 would improve the heat resistance of *B. rapa* remains to be investigated.

### Potential roles of heat-responsive cis-NATs and nat-siRNAs

Eight of the miRNA precursors reported are cis-NATs that overlapp with coding genes. In a previous study, we have identified nine miRNAs that responded to heat stress in *B. rapa*[26]. Among them, miR398a and miR398b were very sensitive to heat stress, and MIR398b precursor was also decreased under heat stress in *B. rapa*. Heat stress induction of miR398 triggers a regulatory loop that is critical for thermotolerance in *Arabidopsis*[35]. In this study, two miR398b precursors were found to overlaps with their adjacent genes. The question arises whether these adjacent genes are involved in the regulation of miR398–mediated thermotolerance.

Heat stress disturbs cellular homeostasis. The accumulation of heat shock proteins under the control of heat stress transcription factors plays a central role in heat resistance [36]. In tobacco plants, genetic engineering of the biosynthesis of glycinebetaine enhances thermotolerance of photosystem II [37], and chloroplastic NAD(P)H dehydrogenase in tobacco leaves alleviates the oxidative damage caused by temperature stress [38]. Three cis-NATs that give rise to heat-responsive nat-siRNA encoded HSP17.4 (small heat shock protein), LHCB3 (a component of the main light harvesting chlorophyll a/b-protein complex of Photosystem II), and NDF4 (a novel subunit of the chloroplast NAD(P)H dehydrogenase complex), meaning that nat-siRNA from those cis-NATs may play important roles in heat resistance.

The present work is based on putative identifications using bioinformatics tools. Northern blots of more nat-siRNAs are necessary to confirm the difference in their accumulation between normal and high temperature. More importantly, the direct evidence is dependent on silencing of nat-siRNA to the target genes in the transgenic plants. Therefore, a great attention should be paid to transfer the nat-siRNAs into crops to verify their usefulness for increase in heat-resistance.

In addition to nucleus, the chloroplast is an important organelle that generates sRNAs. Many members of chloroplast sRNA families are highly sensitive to heat stress, and some silence target genes. To address the roles of heat-responsive nat-siRNAs in plant heat resistance, we plan to compare the gene expression patterns of heat-sensitive Wut with the less heat-sensitive Bre. Analyses of the differentially-expressed nat-siRNAs could provide insight into the molecular mechanism of nat-siRNA-mediated responses. Ultimately, these nat-siRNAs may be useful for genetic improvement of the heat resistance in Chinese cabbage and other important crops.

## Conclusions

In two varieties of *B. rapa*, we identified 1031 cis-NATs, 150 of which gave rise to 4846 nat-siRNAs. Of these, 303 conserved cis-NATs corresponded to 280 cis-NATs in *Arabidopsis*, indicating that 728 novel cis-NATs were *Brassica*-specific. The nat-siRNAs derived from 12 cis-NATs responded to heat stress and most exhibited strand bias. Our work was the first genome-wide analysis of cis-NATs and heat-responsive nat-siRNAs in *B. rapa*; these sRNAs are potentially useful for genetic improvement of heat tolerance of *B. rapa* and other crops.

## Methods

### Plant materials and sequencing

Heading Chinese cabbage (*B. rapa ssp. pekinensis* cv. Bre) and non-heading Chinese cabbage (*B. rapa* ssp. *chinensis* cv. Wut) were used in this study. For RNA sequencing, the seedling (3 weeks old) and inflorescence apices (IA) (2 months old) of Bre and Wut were sampled. Total RNA was extracted with TRIzol (Invitrogen, Carlsbad, CA, USA) and treated with DNase I (Takara, Dalian, China) to remove DNA contamination. Two RNA samples prepared from seedling of Bre and Wut were sent to BGI Shenzhen (Beijing, China) and two RNA samples from IA were sent to macrogen company (Seoul, South Korea) for high throughput cDNA sequencing. For sRNA sequencing, 7-day-old seedlings of Bre were root-inoculated with the bacterial strain (*Erwinia carotovora* subsp. *carotovora*), while seedlings inoculated with sterile water served as mock controls; RNA samples were prepared 2-weeks after inoculation [31]. Three week old seedlings of Wut were exposed to 22°C (NT) or 46°C (HT) for 1 h, with two biological replicates (NT1, NT2, HT1, and HT2) [26]. RNA samples were prepared using the Alternative v1.5 Protocol (Illumina, 2009), and sRNA sequencing was performed using Illumina GAII sequencer and mirVana™ miRNA Isolation Kit (Ambion, Carlsbad, CA, USA).

Small RNAs were isolated using mirVana™ miRNA Isolation Kit (Ambion, Inc), and sRNA sequencing was performed using Illumina GAII sequencer. Although the samples for RNA sequencing and small RNA sequencing were not from the same seedlings, the heat-treated samples for sRNA sequencing were 3-week-old plants of Wut, consistent with the development stages RNA sequencing samples. The RNA-sequences were used to obtain cis-NATs and sRNA annotation, and we focused on comparing the expression of nat-siRNAs between samples with and without heat treatment.

### Alignment and gene annotation

A total of 98291786 paired-end reads were obtained from 4 RNA samples via Illumina sequencing. Using the Tophat-Cufflink pipeline [30], the reads were mapped to the reference genome v1.1 of *B. rapa* by the short read aligner Bowtie, and the transcripts were further assembled by Cufflink with default parameter [30]. Cuffcompare program was used to compare the transcripts with reference gene models (CDS), and the longest of each detected transcript with an extended UTR was annotated as a novel gene models. Conserved and *Brassica*-specific miRNA precursors were locally blasted with *Brasscia* genome and the best hit positions were annotated with the gff3 format. Raw sequence reads of sRNA were parsed to remove 3′-adaptors, and the unique sequences of length 17–36 nt were mapped to the *B. rapa* by SOAP2 software with fewer than two mismatches.

### Identification of cis-NATs and nat-siRNAs

Using the Brassica novel genome annotations containing miRNA precursors and genes with the longest extended UTR in *Brassica*, pairs of genes that overlapped by more than 25 nt, were shorter than 2000 nt, and were transcribed from the opposite DNA strand were regarded as cis-NATs. Small RNAs from overlapping region of cis-NATs that satisfied the following standards were considered to be potential nat-siRNAs: (1) perfect matches with the reference genome; (2) the length of 20–28 nt; and (3) at least 5 reads per 10 million at least in one sRNA library.

### Analysis of strand bias of nat-siRNAs derived from cis-NATs

Nat-siRNA reads derived from the forward and reverse strands of cis-NATs were designated FR and RR, respectively. If the ratio of FR/RR was greater than 2 or less than 0.5 with a *p*-value <0.01 by the Audic and Claverie pairwise test [39], then those nat-siRNAs from cis-NATs were defined as strand biased. The *p*-value was calculated as:

$$p\left(y|x\right)={\left(\frac{N_{2}}{N_{1}}\right)}^{y}\frac{\left(x+y\right)!}{x!y!{\left(1+\frac{N_{2}}{N_{1}}\right)}^{\left(x+y+1\right)}}$$

where *N1* represents the total sRNA read number in the seedlings without heat treatment, while *N2* represents number with heat treatment; *x* represents the abundance of nat-siRNA in seedlings without heat treatment, and *y* represents abundance in seedlings with heat treatment.

### Identification of conserved cis-NATs between *B. rapa* and Arabidopsis

*Arabidopsis* homologs to the global *B. rapa* cis-NATs were selected from the homologous gene table of two species. Genome-wide cis-NATs in *Arabidopsis* were identified using the *Arabidopsis* genome annotation (TAIR version 10) as described above for *B. rapa* The homologous cis-NATs were considered to be conserved cis-NATs (Additional file 3: Table S5).

### Differential expression analysis of heat-responsive nat-siRNAs

The Bayesian test method was used to determine the expression differences between the two temperature conditions (NT and HT) by compariing tag counts generated from digital expression analyses [39]. In both replicates, the ratios of total reads of nat-siRNAs from cis-NATs in the two conditions (HT1/NT1 and HT2/NT2) were more than 2 (or less than 0.5) and the *p*-value <0.01, so these differences were considered significant.

### Real-time PCR

Total RNA was extracted with TRIzol (Invitrogen) and treated with DNase I (Takara) to remove DNA contamination. Approximately, 4 μg of RNA was used for reverse transcription with oligo-dT primers. Real-time PCR was performed using specific pairs of primers (Additional file 7: Table S7). The comparative threshold cycle (Ct) method was used to determine relative transcript levels in real-time PCR (MyiQ2, Two-Colors Real-time PCR Detection System, Bio-Rad, Hercules, CA, USA). *BrcACT4* of *B. rapa*, which is homologous to *Arabidopsis ACT4* encoding actin protein, was used as an internal control. Three biological replicates and three technological replicates were performed.

### Small RNA purification and Northern blot

Small RNA purification was performed referring to protocol of Hamilton and Baulcombe [40] with minor modifications. Coding sequence of Bra018216 was amplified with primer pairs Bra018216-8S and Bra018216-337A (Additional file 7: Table S7), and single–stranded RNA probes were transcribed from antisense transcripts of Bra018216 using T7 RNA polymerase with digoxin-labeled uridine triphosphate (Dig-11-dUTP). 30–50 μg RNA was separated on 19% polyacylamide denaturing gels, and transformed to Hybond membrane for 14 h at 28 mA. After cross-linking for 4 min with UV irradiation and 80°C for 2 h, the Hybond membrane was hybridized with digoxin-labeled probe at 37°C overnight, and was washed twice at 37°C with 2× SSC and 0.2% SDS for 15 min. Then, the membrane was incubated with blocking reagent for 30 min, and reacted with Anti-Digoxigenin-AP Fab fragment, and again was washed with 2× SSC and 0.2% SDS for 15 min. At last, the membrane was equilibrated with equilibration buffer, soaked in CDP-Star solution, and exposed to x-ray file (Fuji film). Blots were also probed with a biotin-marked DNA probe complementary to U6 as an internal control, using the Thermo scientific Kit.

### Availability of supporting data

The raw data has been submitted to the NCBI Sequence Read Archive (SRA) (http://www.ncbi.nlm.nih.gov/sra). The SRA accessions of 4 RNA sequencing experiments are SRX323438, SRX323448, SRX323442 and SRX323460.

## Abbreviations

B. rapa: *Brassica rapa*; CDS: Contained coding sequence; cis-NATs: cis-natural antisense transcripts; Ecc: *Erwinia carotovora* subsp. *carotovora*; HT: High temperature; Intronic miRNAs: Intron-derived miRNAs; miRNAs: microRNAs; nat-siRNAs: NAT-derived small interfering RNAs; NATs: Natural antisense transcripts; pre-miRNAs: miRNA precursors; NT: Normal temperature; RP10M: Reads per 10 million; sRNAs: small RNAs; ta-siRNAs: trans-acting small interfering RNAs; UTR: Un-translated region.

## Competing interests

The authors declare that they have no competing interests.

## Authors’ contributions

YX carried out the sampling for RNA sequencing, assembly of transcripts, and identification of cis-NATs and nat-siRNAs, and wrote the manuscript. YJ and LXR performed validation experiments. LXX arranged the sRNAs libraries in SQL database. SCB took part in sRNA sequencing. YJ and WFJ revised the manuscript, and HYK designed the whole research project and improved the manuscript. All authors read and approved the final manuscript.

## Supplementary Material

Additional file 1: Table S1 Overview of four RNA-seq dataset. Click here for file

Additional file 2: Table S2 Gene model annotation of miRNA precursors in *B. rapa.* Click here for file

Additional file 3: Table S5 Cis-NAT Homology between B. rapa and Arabidopsis. Click here for file

Additional file 4: Table S3 Cis-NAT pairs that nat-siRNAs are mapped to in Bre. Click here for file

Additional file 5: Table S4 Cis-NAT pairs that nat-siRNAs are mapped to in Wut. Click here for file

Additional file 6: Table S6 Nat-siRNAs from Wut with normal and high temperature. Click here for file

Additional file 7: Table S7 Primers used for real-time PCR of cis-NATs. Click here for file
